# VarI-SIG 2014 - From SNPs to variants: interpreting different types of genetic variants

**DOI:** 10.1186/1471-2164-16-S8-I1

**Published:** 2015-06-18

**Authors:** Yana Bromberg, Emidio Capriotti

**Affiliations:** 1 Department of Biochemistry and Microbiology, Rutgers University, 08901 New Brunswick (NJ), USA; 2 Department of Genetics, Rutgers University, 08854 Piscataway (NJ), USA; 3 Division of Informatics, Department of Pathology, University of Alabama at Birmingham, 619 19th St. South, 35249 Birmingham (AL), USA; 4 Department of Clinical and Diagnostic Sciences, University of Alabama at Birmingham, 619 19th St. South, 35249 Birmingham (AL), USA; 5 Department of Biomedical Engineering, University of Alabama at Birmingham, 619 19th St. South, 35249 Birmingham (AL), USA

## Introduction

The decreasing cost of high-throughput sequencing is still rapidly increasing the number of known genetic variants [1,2]. For example, the size of the dbSNP database [3] has nearly doubled over the past years to 110 million human single nucleotide polymorphisms and short genomic variants. Unfortunately the characterization, annotation, and interpretation of these variants are still lagging. In particular, the interpretation of genetic variants and their implication in disease is one of the major challenges in personalized medicine [4-6].

The 4^th ^edition of the **Var**iant **I**nterpretation **S**pecial **I**nterest **G**roup (VarI-SIG, formerly SNP-SIG) meeting [7-9] was held on July 12 at the ISMB 2014 in Boston (MA). The central meeting themes were "Annotation and prediction of structural/functional impacts of coding variants" and "Genetic variants as effectors of change: disease and evolution". The VarI-SIG is organized as a venue for the development of a research network of scientists, necessary for facilitating the exchange of ideas and establishing new collaborations. This year's meeting attracted over 100 participants, with seven research talks and five presentations from the leading scientists of the field.

## Manuscript submission and review

This year we received 11 submissions to the VarI-SIG special issue. All submissions were by-invitation on the basis of talks/interactions that took place at the meeting. In addition to editorial evaluations, all manuscripts were assessed by at least two reviewers from a panel of 22 experts in the field (see Acknowledgements). To maintain reviewer anonymity, the four reviewers of submissions in which the editors are co-authors remain un-named. After 2 rounds of review 9 of the 11 manuscripts were accepted for publication in this special issue.

The articles presented in this SIG proceedings issue focus on annotating genomic variants in coding [10] and non-coding regions [11], identifying phenotypic impacts of genetic and epigenetic variations [12-14], characterizing biological basis of Mendelian diseases [15] and interpreting the cancer genome [16], detecting positive selection in regulatory regions [17], and identifying geographical origins of individuals [18].

[The complete program of VarI-SIG meeting 2014 with presentation and poster abstracts is available at http://varisig.biofold.org/2014/docs/vari-sig-2014-programme.pdf]

## Further developments

This year we welcome to the VarI-SIG organizing committee Dr. Hannah Carter, Assistant Professor, Division of Medical Genetics, UCSD. Together, we are working on the organization of the next VarI-SIG meeting that will be held in the context of the ISMB/ECCB 2015 (Dublin, Ireland; July 11, 2015). Further information about the meeting is available on our website (http://varisig.biofold.org).

In collaboration with ISMB organizers, we are also promoting the formation of VarI-COSI (Variant Interpretation Community of Special Interest). VarI-COSI is community aimed at sharing relevant information, discussing ideas, and providing training and support networks in the field of genomic interpretation. We are still in the initial stages of setting up our COSI presence on the web and look forward to input and participation from the variation interpretation community.

## Competing interests

The authors declare they have no conflict of interests in relation to this VarI-SIG 2014 special issue article.

## Authors' contributions

YB and EC wrote the manuscript. Both authors read and approved the manuscript.
